# Hospitalization at the end of life in patients with multiple myeloma

**DOI:** 10.1186/s12885-021-08079-x

**Published:** 2021-03-31

**Authors:** Saqib Abbasi, John Roller, Al-Ola Abdallah, Leyla Shune, Brian McClune, Douglas Sborov, Ghulam Rehman Mohyuddin

**Affiliations:** 1 Department of Hematological Malignancies and Cellular Therapeutics, Kansas University Medical Center, Kansas City, USA; 2 Division of Hematology and Hematologic Malignancies, University of Utah, Salt Lake City, USA

**Keywords:** Multiple myeloma, Palliative care, Hospice, Death, National inpatient sample, Inpatient, Hospital

## Abstract

**Background:**

Despite advances in treatment, multiple myeloma (MM) remains incurable and results in significant morbidity and mortality. Further research investigating where MM patients die and characterization of end-of-life hospitalizations is needed.

**Methods:**

We utilized the National Inpatient Sample (NIS) to explore the hospitalization burden of MM patients at the end of their lives.

**Results:**

The percent of patients dying in the hospital as a percent of overall MM deaths ranged from 54% in 2002 to 41.4% in 2017 (*p* < 0.01). Blood transfusions were received in 32.7% of these hospitalizations and infections were present in 47.8% of patients. Palliative care and/or hospice consultations ranged from 5.3% in 2002 to 31.4% in 2017 (*p* < 0.01).

**Conclusion:**

Our study demonstrates that patients with MM dying in the hospital have a significant requirement for blood transfusions and have a high infection burden. We also show that palliative care and hospice involvement at the end of life has increased over time but remains low, and that ultimately, inpatient mortality has decreased over time, but MM patients die in the hospital at a higher rate than the general population.

**Supplementary Information:**

The online version contains supplementary material available at 10.1186/s12885-021-08079-x.

## Introduction

Due to the routine incorporation of highly effective agents such as proteasome inhibitors, immunomodulatory drugs and anti CD38 targeting drugs, there has been a dramatic improvement in survival for patients with myeloma [1–3].. However, even with these advances in management, MM is projected to cause 13,000 deaths in the United States annually [4].

Despite these significant number of deaths, there is limited data on the manner and environment in which MM patients die. Previous work focusing mostly on early mortality has demonstrated that renal failure and infection are leading causes of death [5]. However, further research on mortality is needed including examining where MM patients primarily die, whether it be in the hospital or at home, and use of supportive care services including transfusions, antibiotics, and specialty services such as palliative care or hospice.

To better understand these questions and provide better context to the mortality of MM patients, we utilized the National Inpatient Sample (NIS) to explore the hospitalization burden of MM patients at the end of their lives.

## Methods

### Data source

The NIS is a database compiled by the Agency for Healthcare Research and Quality (AHRQ) annually since 1988. The number of States participating in the NIS has grown from 8 in the first year to 47, plus the District of Columbia, currently. Approximately 20% of national admissions are tracked and weighted estimates are provided regarding the total number of hospitalizations [6]. Unweighted, it contains data from more than 7 million hospital stays each year including patient demographics, primary and secondary diagnoses, procedures, length of stay, and disposition. Each observation within the database comprises a unique hospitalization. However, the NIS does not identify individual patients and recurrent hospitalizations are recorded as distinct observations [7]. It also does not capture outpatient encounters, observation-only stays, or stays in long-term acute care hospitals or rehabilitation centers.

### Study population

**Fig. 1:**
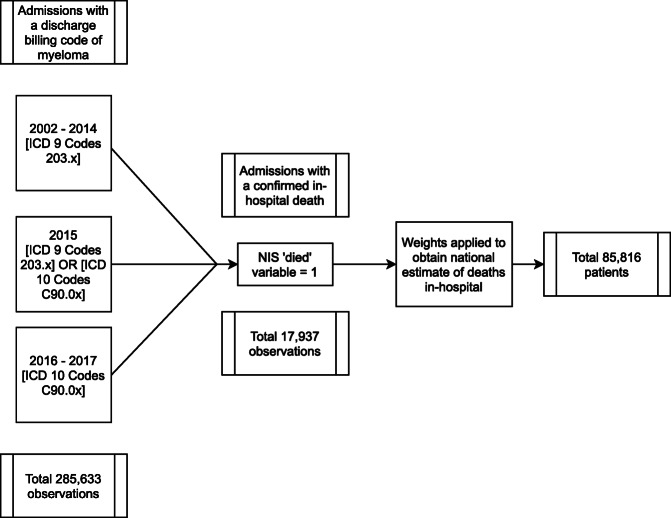
Flowchart Outlining Selection of Patients

Using the NIS, all patients discharged from a hospital setting with an International Classification Code (ICD) for multiple myeloma from the years 2002 to 2017 were collected. Patients who were noted to die in hospital were then sub-selected (Fig. 1). ICD 9 and ICD 10 codes were then used to gain insight into trends in transfusions, infectious complications, and costs for admission. For demographics, a Chi-square test was utilized to compare variables of gender, comorbidity and race between years 2006 and 2017, whereas a pooled t-test was used for age. All ICD codes utilized are shown in the supplement. Overall annual number of deaths for MM in the United States was obtained from publicly available reports from the Centers for Disease Control (CDC) and Prevention and the National Cancer Institute (NCI).

### Outcomes

The primary endpoint of our study was to determine the percentage of patients with multiple myeloma dying in the hospital and investigate these trends from 2002 to 2017. Secondary endpoints included palliative care/hospice consultation during this time period and prevalence of infection, need for transfusions and charges related to hospitalization. Infection frequency was identified using the Clinical Classification Software that allows for clustering patient diagnoses and procedures into clinically meaningful categories. Demographic information on age, sex, race and comorbidity were collected and we also ascertained whether or not the hospitalization in question was associated with treatment of MM by using ICD codes for chemotherapy administration.

## Results

During the time period from 2002 to 2017, the CDC reported a total of 180,924 MM deaths ranging from 10,913 in 2002 to 12,322 in 2017. The NIS identified an estimated national total of 1,446,809 hospitalizations for MM during this time period. Amongst these, an estimate of 85,816 hospitalizations resulted in deaths. Thus, 6.3% of all hospitalizations for MM patients resulted in death. During our study time period, 47.4% of all deaths related to MM in the United States occurred in the hospital. The percent of patients with MM dying in the hospital as a percent of overall myeloma-related deaths ranged from 54% in 2002 to 41.4% in 2017, *p* < 0.01.

### Proportion of hospitalizations and mortality related to chemotherapy

There were a total of 54,357 admissions for chemotherapy from 2002 to 2017, out of which 1.01% resulted in mortality, for a total of 547 deaths. Thus, only 547 of the 85,816 of the overall deaths were in hospitalizations in which chemotherapy was administered, representing only 0.64% of all the deaths during this time period.

### Demographic trends

Demographic trends of MM patients who died during hospitalizations from 2006 to 2017 are outlined in Table 1. Data prior to 2006 for comparison as the information in the dataset for these years could not accurately code for kidney function. The average age of death was seen to be similar at 71.49 years in 2006 vs. 72.37 years in 2017 (*p* < 0.077). A slightly higher proportion of female deaths compared to males at 57.9% vs. 42.1% was seen in 2017 vs. 2006 when the proportion was 53.5% vs. 46.5% (*p* < 0.04). Upon examining the ethnic background of these patients, black patients had an increased proportion in the 2017 population at 22.04% of all hospitalizations leading to death vs. 14.30% in 2006 (*p* < 0.005), however, a large number of patients had missing data in 2002 (Table 1).

**Table 1:** Demographics of Studied Population

Demographics table					
Year	2006		2017		*P*-value
Demographic	%	#	%	#	
Age	71.49 (mean)		72.37 (mean)		0.077
Gender					
Male	46.5%	527	42.1%	430	0.04
Female	53.5%	606	57.9%	591	
CKD stage V	1.20%	14	0.90%	9	0.425
End Stage Renal Disease	14.80%	168	12.70%	130	0.160
Hypertension	44%	503	12%	127	< 0.001
Congestive Heart Failure	33.00%	374	33%	379	0.924
Race					
White	49.96%	566	57.00%	582	0.005
Black	14.30%	162	22.04%	225	
Hispanic	5.65%	64	9.50%	97	
Asian or Pacific Islander	1.85%	21	2.74%	28	
Native american	0.71%	8	0.49%	5	
Other	1.85%	21	4.41%	45	
Missing	25.68%	291	3.82%	39	
Total		1133		1021	

### Blood transfusion usage

We assessed blood transfusion dependency in the hospitalization leading to death. Blood transfusions were received (32.7%) in 39,393 of the 85,816 admissions leading to death.

### Infection burden

A total of 41,063 infections were identified amongst the 85,816 hospitalizations leading to death (47.8%).

### Palliative care/hospice involvement

**Fig. 2:**
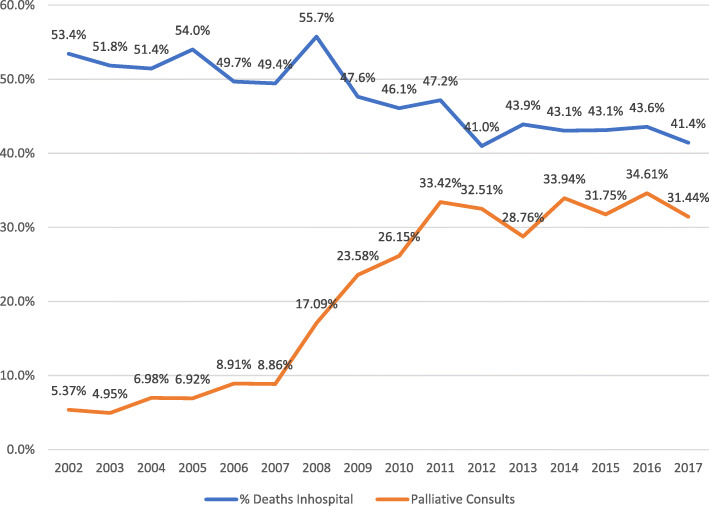
Trends of Deaths and Palliative Consults for MM Hospitalized Patients

We analyzed palliative care/hospice involvement during the hospitalization leading to death from 2002 to 2017. Palliative care/hospice was consulted in 67 of the 1260 hospitalizations in 2002 (5.3%), and increased in 2017, whereas a palliative care/hospice consultation was seen in 321 out of the 1021 hospitalizations in (31.44%) (*p* < 0.01). Figure 2 highlights the trend of these consultations.

### Cost

We evaluated the charge of each MM hospitalization leading to death. Median cost of the hospitalization leading to death after adjustment for inflation increased over time from $62, 202 in 2002 to $150,451 in 2017, *p* < 0.001.

## Discussion

Our study is the first to use a nationwide database to compare with publicly available mortality data to provide an estimate of the overall percentage of deaths from MM occurring in the hospital. Our study shows that despite advances in MM therapy and supportive care, greater than 40% of patients with MM continue to die in the hospital as of 2017, although there has been a reassuring decrease since 2002. This trend mirrors data for overall deaths in the hospital in the US, which has shown that 48.0% of deaths from all causes occurred in hospital in 2000 but decreased to 35.1% in 2014 [8]. Our analysis thus demonstrates that patients with MM continue to die in the hospital at greater rates than the general population, findings that have also been demonstrated for other hematological malignancies such as leukemia [9].

As only a small proportion of hospitalizations were related to chemotherapy, which are often planned admissions with a therapeutic intent, our data reflects that the vast majority of hospitalizations resulting in death for MM patients reflect unplanned admissions. This paper did not specifically account for deaths in the hospitalization for a stem cell transplantation amongst overall inpatient deaths. However, we previously have interrogated the National Inpatient Sample to evaluate for autologous transplantation related hospitalizations, and the total weighted number of inpatient admissions for stem cell transplantation among MM patients was 47,253 from 2002 to 2014, with an inpatient mortality ranging from 1.8% in 2002 to 0.7% in 2014. Thus only a small minority of deaths in the hospital from our dataset would have been related to a hospitalization for transplantation [10].

We also observed an increase in the proportion of African Americans dying in the hospital over time, which is consistent with other studies of African American patients with malignancy that have higher shown likelihood of dying in acute care setting [11–13]. We would however draw caution in interpreting this finding as inferences on race from the National Inpatient Sample is problematic due to a high proportion of patients with “missing” race in earlier years, and that race is variably and inconsistently reported between states.

Our analysis demonstrates significant transfusion requirements and infections seen at the end of life with MM patients. We observed that infections were identified in a notable 45% of MM hospitalizations leading to death, and almost 40% of these hospitalizations required blood transfusions. This significant rate of infection is consistent with previous data that has demonstrated infection as a major driver of early MM death [5]. Although our dataset cannot differentiate early vs. late MM mortality, our data demonstrates that infection plays an important role throughout its disease course [6, 14]. Although prophylactic antibiotics are now increasingly used in induction [15], their value and use later in the disease course is worthy of further attention.

The high requirement for transfusions for MM patients seen at the end of life likely led to a delay in utilization of hospice services. It is not possible to infer the exact reason for hospitalization using the National Inpatient Sample but given the high burden of transfusions in our dataset, it is reasonable to infer that a significant proportion of hospitalizations were due to transfusion requirements. Lack of access to transfusions is a well-recognized barrier to enrollment on hospice. Hospice agencies often do not have the financial resources to provide these transfusions because most payers, such as Medicare, reimburse at a fixed daily rate for patients, irrespective of service types provided [16, 17]. However, allowing transfusions during hospice may be more cost effective for overall health system as it may increase hospice enrollment, which has been associated with lower rates of inpatient death, ICU admission in the last 30 days of life, and chemotherapy use in the last two weeks of life [17–21].

We also found charges increased significantly during our time period, doubling even when adjusted for inflation. Allowing for earlier access to transfusions and palliative care/hospice can prevent many of these admissions, and lead to less health-care resource utilization for patients with MM. Earlier work using Medicare data has shown that hospice usage has been increasing for patients with MM, increasing from 28.5% in 2000 to 56.5% by 2013, with only a slight decrease in aggressive end-of-life care (59.2% in 2000 to 56.7% in 2013, 21]. This information taken together with our data highlights the significant burden of patients with MM dying in the hospital and identifies the need for dramatic improvement in this area.

There are several important limitations to our study. It did not include information on outpatient encounters or stays in rehabilitation centers or acute care hospitals, and thus palliative care/hospice usage as an outpatient cannot be ascertained from our dataset. In addition, it did not include clinical data that is specific to cancer such as performance status, cytogenetic risk, stage of disease or laboratory data. In addition, our analysis used aggregate patient data instead of individual patient data so variable effects on particular patient subgroups could not be analyzed.

In summation, our study demonstrates a reassuring decrease in inpatient mortality for patients with MM over time, although patients continue to die in the hospital at greater rates than the general population. Palliative care and hospice involvement at the end of life has increased over time but remains low. Patients with MM dying in the hospital have a significant requirement for blood transfusions and have a high infection burden. Facilitating access to blood transfusions as part of hospice can increase hospice enrollment and decrease the burden and cost of these hospitalizations. Concerted multi-disciplinary efforts and further research in this area are needed in order to further improve our approach towards the end of life in patients with MM.

## Supplementary Information

**Additional file 1 **: **Table S1.** Utilized ICD Codes.

## Data Availability

The source material for this study is the National Inpatient Sample, which is publicly available data at https://www.hcup-us.ahrq.gov/nisoverview.jsp. The corresponding author Dr. Mohyuddin may be contacted for additional information.

## References

[1] Turesson I, Bjorkholm M, Blimark CH, Kristinsson S, Velez R, Landgren O (2018). Rapidly changing myeloma epidemiology in the general population: increased incidence, older patients, and longer survival. Eur J Haematol.

[2] Mikhael J, Ismaila N, Cheung MC, Costello C, Dhodapkar MV, Kumar S, Lacy M, Lipe B, Little RF, Nikonova A (2019). Treatment of multiple myeloma: ASCO and CCO joint clinical practice guideline. J Clin Oncol.

[3] Kumar SK, Dispenzieri A, Lacy MQ, Gertz MA, Buadi FK, Pandey S, et al. Continued improvement in survival in multiple myeloma: changes in early mortality and outcomes in older patients. Leukemia. 2014;28(5):1122–8. 10.1038/leu.2013.313. 10.1038/leu.2013.313 PMC4000285 24157580

[4] National Cancer Institute. Myeloma - Cancer Stat Facts. Surveillance, Epidemiology, and End Results (SEER) Program. https://seer.cancer.gov/statfacts/html/mulmy.html. Accessed 20 Oct 2020.

[5] Augustson BM, Begum G, Dunn JA, Barth NJ, Davies F, Morgan G, Behrens J, Smith A, Child JA, Drayson MT (2005). Early mortality after diagnosis of multiple myeloma: analysis of patients entered onto the United Kingdom Medical Research Council trials between 1980 and 2002—Medical Research Council adult Leukaemia working party. J Clin Oncol.

[6] Blimark C, Holmberg E, Mellqvist U-H, Landgren O, Björkholm M, Hultcrantz M, Kjellander C, Turesson I, Kristinsson SY (2015). Multiple myeloma and infections: a population-based study on 9253 multiple myeloma patients. Haematologica.

[7] Khera R, Krumholz HM. With Great Power Comes Great Responsibility: Big Data Research From the National Inpatient Sample. Circ Cardiovasc Qual Outcomes. 2017;10(7):e003846. 10.1161/CIRCOUTCOMES.117.003846. 10.1161/CIRCOUTCOMES.117.003846 PMC5728376 28705865

[8] National Vital Statistics System. Underlying cause of death data**,** 2000–2018**.** [https://wonder.cdc.gov/ucd-icd10.html.]

[9] Mohyuddin GR, Abbasi S, Ripp J, Singh A, Kambhampati S, McClune B (2020). Patients with leukemia dying in the hospital: results of the national inpatient sample and a call to do better. Leuk Lymphoma.

[10] Abbasi S, Britt A, McClune B, Shune L, Kambhampati S, Mohyuddin GR (2020). Inpatient hospitalization's associated cost and mortality in myeloma patients undergoing autologous stem cell transplant: a 13-year analysis of the National Inpatient Sample. Leuk Lymphoma.

[11] Sedhom R, Kuo PL, Gupta A, et al. Changes in the place of death for older adults with cancer: reason to celebrate or a risk for unintended disparities? J Geriatr Oncol. 2020;S1879-4068(20)30480-X. 10.1016/j.jgo.2020.10.008. [published online ahead of print, 2020 Oct 26] 10.1016/j.jgo.2020.10.008 PMC8428799 33121909

[12] Chino F, Kamal AH, Chino J, LeBlanc TW (2019). Disparities in place of death for patients with hematological malignancies, 1999 to 2015. Blood Adv.

[13] Chino F, Kamal AH, Leblanc TW, Zafar SY, Suneja G, Chino JP (2018). Place of death for patients with cancer in the United States, 1999 through 2015: racial, age, and geographic disparities. Cancer.

[14] SAVAGE DG, LINDENBAUM J, Garrett T (1982). Biphasic pattern of bacterial infection in multiple myeloma. Ann Intern Med.

[15] Drayson MT, Bowcock S, Planche T, Iqbal G, Pratt G, Yong K, Wood J, Raynes K, Higgins H, Dawkins B, Meads D, Hulme CT, Monahan I, Karunanithi K, Dignum H, Belsham E, Neilson J, Harrison B, Lokare A, Campbell G, Hamblin M, Hawkey P, Whittaker AC, Low E, Dunn JA, TEAMM Trial Management Group and Trial Investigators (2019). Levofloxacin prophylaxis in patients with newly diagnosed myeloma (TEAMM): a multicentre, double-blind, placebo-controlled, randomised, phase 3 trial. Lancet Oncol.

[16] Medicare Program; FY 2021 Hospice Wage Index and Payment Rate Update [https://www.federalregister.gov/documents/2020/08/04/2020-16991/medicare-program-fy-2021-hospice-wage-index-and-payment-rate-update]. Accessed 20 Oct 2020.

[17] LeBlanc TW, Egan PC, Olszewski AJ (2018). Transfusion dependence, use of hospice services, and quality of end-of-life care in leukemia. Blood.

[18] Egan PC, LeBlanc TW, Olszewski AJ (2020). End-of-life care quality outcomes among Medicare beneficiaries with hematologic malignancies. Blood Adv.

[19] Brooks GA, Li L, Uno H, Hassett MJ, Landon BE, Schrag D (2014). Acute hospital care is the chief driver of regional spending variation in Medicare patients with advanced cancer. Health Aff (Millwood).

[20] Odejide OO, Cronin AM, Earle CC, Tulsky JA, Abel GA (2017). Why are patients with blood cancers more likely to die without hospice?. Cancer.

[21] Odejide OO, Li L, Cronin AM, Murillo A, Richardson PG, Anderson KC, Abel GA (2018). Meaningful changes in end-of-life care among patients with myeloma. haematologica.

