# Supplementary material for: Hospitalization at the end of life in patients with multiple myeloma
Source: BMC Cancer. 2021 Mar 31;21:339. doi: 10.1186/s12885-021-08079-x (PMC8011131; doi:10.1186/s12885-021-08079-x)
Supplement: Supplementary file 1 — Additional file 1 : Table S1. Utilized ICD Codes. [file 12885_2021_8079_MOESM1_ESM.docx]

Supplementary Table 1:Utilized ICD Codes

| Condition | ICD 9 | ICD 10 |
| --- | --- | --- |
| Multiple Myeloma | 203.0x | C90.0x |
| Blood Transfusion | 99.0x | 30233Nx, 30233Rx, 30230Nx, 30230Rx, 30243Nx, 30243Rx, 30240Nx, 30240Rx |
| Infection: |  |  |
| Pulmonary  Urinary  Gastrointestinal | 460.x-466.x, 480.x-488.x  590.x, 595.x, 597.x  001.x-009.x | J09.x-J18.x  N10.x, N30.x, N34.x, N39.x |
|  |  | A00.x-A09.x |
| Palliative Care | V66.7 | Z51.5 |
| End stage renal disease | 585.6 | N18.6 |
| Hypertension | 401.x-405.x | I10.x-I16.x |
| Congestive Heart Failure | 428.x | I50.x |
